# Defective Soil for a Fertile Seed? Altered Endometrial Development Is Detrimental to Pregnancy Success

**DOI:** 10.1371/journal.pone.0053098

**Published:** 2012-12-31

**Authors:** Jemma Evans, Natalie J. Hannan, Cassandra Hincks, Luk J. F. Rombauts, Lois A. Salamonsen

**Affiliations:** 1 Prince Henry’s Institute of Medical Research, University of Melbourne, Melbourne, Victoria, Australia; 2 Department of Zoology, University of Melbourne, Melbourne, Victoria, Australia; 3 Obstetrics and Gynaecology, Monash University, Melbourne, Victoria, Australia; 4 Monash IVF, Melbourne, Victoria, Australia; Queen's University, Canada

## Abstract

**Background:**

Synchronous development of the endometrium (to achieve a receptive state) and of the embryo is essential for successful implantation and ongoing pregnancy. Endometrial receptivity exists only for a finite time in a menstrual cycle and the endometrium is refractory to embryo implantation outside of this window. Administration of hormones to stimulate multifollicular development within the ovary, integral to the majority of assisted reproduction (ART) protocols, dramatically alters the hormonal milieu to which the endometrium is exposed versus normal menstrual cycles. Endometrial maturation may be profoundly affected by this altered endocrine environment.

**Aim:**

Compare endometrial histology in fertile women, fertile women undergoing hormonal stimulation for oocyte donation and infertile women undergoing fresh embryo transfers in an ART cycle with further comparisons between women who did or did not become pregnant. Examine the presence of leukocytes and markers of endometrial maturation.

**Methods:**

Endometrial histology was examined by hematoxylin and eosin staining with a semi quantitative scoring method developed to compare histological appearance of tissues. The presence of leukocytes and developmental markers was examined by immunohistochemistry and scored.

**Results:**

Endometrial histology was dramatically altered upon stimulation for ART. However, those women who became pregnant presented with significantly less alterations in histological endometrial maturation. Numbers and activation status of leukocyte populations were also altered within the endometria stimulated for ART, with neutrophils undergoing degranulation, usually observed only pre-menstrually.

**Conclusion:**

We propose that such developmental changes render the endometrium hostile to the embryo and that modifications to ART protocols should be considered to take account of the requirement for endometrial receptivity and hence increase pregnancy rates.

## Introduction

Implantation of the embryo into the endometrium is an essential step to a healthy ongoing pregnancy. A developing embryo can only implant during the short time in each cycle when the endometrium is receptive (the ‘window of implantation’ [1]). During this receptive phase the endometrium secretes a host of ‘pro-implantation’ factors and undergoes plasma membrane transformation in preparation for implantation of an embryo [1]. In conception cycles there is an embryo-maternal dialogue, conducted at least in part via secretions from both the blastocyst and the maternal endometrial epithelium [2], [3], [4], [5], [6], [7].

During a natural menstrual cycle the endometrium becomes receptive under the influence of progesterone following estrogen priming. In such natural cycles, receptivity is achieved gradually during fertilisation and passage of the developing embryo down the Fallopian tubes towards the uterine cavity, so that developmental synchrony occurs in a timely manner. In assisted reproduction cycles however, the blastocyst is transferred into the uterine cavity when it reaches an appropriate stage of development, without assessment of the synchronicity between maternal tissue and the conceptus.

To date, considerable research energy has been focused on the hormonal regimes administered in assisted reproductive treatment (ART) cycles to achieve an optimal number of high quality eggs for fertilisation. However, pregnancy rates resulting from fresh embryo transfers still hover around 30% [8] for single embryo transfers, in spite of considerable improvement in egg selection and quality. Therefore, a major objective is now to improve endometrial receptivity. This is particularly relevant in light of recent studies demonstrating higher pregnancy rates with frozen-thawed embryos transferred into natural cycles [9], [10].

Endometrial receptivity in normal menstrual cycles is still not well defined at a molecular level [1]. Current effort is directed towards defining markers of endometrial receptivity during the putative window of implantation [3], [4], [6], [11], [12], [13], [14], [15], [16], which lasts for approximately 4 days of each menstrual cycle spanning days 6–10 post ovulation [17]. Outside of these 4 days the endometrium represents a hostile environment for the implanting blastocyst. The ART endometrium however, may present unique challenges to the transferred embryo. It is well documented that the hormonal environment, which determines endometrial development, is significantly altered during ART cycles [18], [19], [20], [21], [22], [23], [24], and the window of receptivity may therefore be altered (in terms of timing) or even absent.

Histological, immunological, transcriptomic, proteomic and secretomic studies have demonstrated that the ART endometrium presents a different environment to the blastocyst compared with normally cycling endometrium at the same stage of the menstrual cycle [5], [20], [21], [23], [25], [26], [27], [28], [29]. Endometrial biopsies obtained during ART cycles, using gonadotropin releasing hormone (GnRH) antagonist or GnRH agonist protocols, display histological advancement and altered gene expression (generally at the time of embryo pickup, hCG+2) compared with biopsies from normal women on the equivalent post ovulation day (POD) 2 (LH+2), according to the gold standard Noyes criteria [20].

Previous histological studies of ART endometria have lacked descriptive microscopic images for the cellular changes described and specific markers for developmental changes. Furthermore, the focus has been on a single ART protocol compared with fertile endometria taken at the equivalent stage of the natural cycle, making it difficult to directly compare protocol effects. Therefore, the aims of this study were to present **a)** a comprehensive comparison of the fertile non-stimulated endometrium with stimulated endometria from fertile (donor cycles) and infertile women, **b)** comparisons between stimulation protocols (GnRH agonist vs GnRH antagonist), and **c)** comparisons between subjects who subsequently became pregnant and those who did not. A number of parameters were examined: key histological features of the endometrium, the presence of developmental markers (progesterone receptor (PR) and prolactin), the abundance of specific immune cell populations and the localisation and appearance of blood vessels.

We demonstrate significantly altered endometrial histology in those women who did not become pregnant, with fewer developmental alterations in those who did become pregnant. We also demonstrate altered leukocyte numbers and the presence of activated neutrophils in the endometria of those women who did not achieve pregnancy.

## Materials and Methods

### Ethics Statement

Ethical approval was obtained from Institutional Ethics Committees at Southern Health and Monash Surgical Private Hospital for all tissue collections. Written informed consent was obtained from all subjects.

### Tissue Collection and Patient Details

This is a retrospective study comparing the endometrial histology of normal fertile women at LH+2 (peak levels measured in urine samples) with women stimulated for ART or oocyte donation at hCG+2 (oocyte pick up). Patients with uterine abnormalities such as leiomyomas, endometrial polyps, or who had received steroid hormone therapy (other than that associated with current treatment, described below) in the last 6 months were excluded. We studied 57 women undergoing in vitro fertilization (IVF), intracytoplasmic sperm injections (ICSI) or oocyte donation cycles between August 2006 and August 2008. We did not apply a cut off for age, BMI, parity, live births or previous ART cycles (Table 1). Cumulative FSH dose, peak estrogen and numbers of oocytes collected were also recorded (Table 1). No differences in embryo quality were noted with 1–2 embryos transferred per cycle.

**Table 1:** Patient demographics.

	Fertile	Donor agonist	Antagonist	Agonist not-pregnant	Agonist pregnant
number of subjects	9	14	14	16	13
age	35.8±3.5(26.3–50)	36.1±0.8^a^(30.3–43.2)	39.4±0.8^b^(32.4–43.8)	34.9±1^a^(28.8–41.5)	35.6±1^a^(28.6–39.5)
BMI (kg/m^2^)	26.1±2.1(17–32)	26.7±1.4^a^(20.5–38.5)	23.6±1.1^b^(19.6–32.4)	27.6±1.5^a^(20.5–39.9)	26.0±1.2(20.0–35.4)
collected oocytes	n/a	10.9±1.2(4–17)	8.6±1.2^a^(4–18)	8.6±1.2^a^(2–20)	14.6±2.7^b^(6–45)
peak estrogen(pg/ml)	n/a	3677±644(407–7945)	2578±379^a^(1350–6046)	2756±526^a^(583–8803)	5556±1347^b^(2115–17854)
cycle number	n/a	2.1±0.6^a^(1–8)	5.5±1.5^b^(1–19)	4.9±1.3^b^(1–21)	2.9±0.9(1–11)
cumulative FSH (IU)	n/a	2500±385(1200–5400)	3161±330(1250–5400)	2858±276(1350–4500)	2429±299(1200–4950)
parity	1.5±0.6(0–4)	1.8±0.3(0–4)	2±0.4(0–6)	0.8±0.2(0–2)	0.9±0.3(0–3)
live births	1.2±0.7(0–4)	1.5±0.3(0–3)	0.7±0.2(0–2)	0.3±0.1(0–1)	0.4±0.1(0–1)

The average age, BMI, number of oocytes collected, peak estrogen levels, cycle number, cumulative FSH, parity and previous live births were calculated for normally cycling fertile women (control subjects), fertile donor women undergoing GnRH agonist protocol (donor agonist), infertile women undergoing GnRH antagonist protocol (antagonist), infertile women undergoing GnRH agonist protocol who did not become pregnant (agonist non pregnant) and infertile women undergoing GnRH agonist protocol who became pregnant in that cycle (agonist pregnant). Data are presented as mean ± SEM with the range presented in brackets. a is significantly different from b, p<0.05.

In the GnRH agonist group (GnRH-a, n = 29) oral contraceptive pill (OCP) was commenced between day 1 and day 7 of the preceding cycle for at least 21 days, followed by administration of the GnRH agonist (Synarel, 0.4 mg per day) 15 days after commencing OCP. After 10–14 days of GnRH agonist, ovarian stimulation was commenced with 125–225IU recombinant follicle stimulating hormone (FSH) daily (dose used dependent on age, BMI>30 kg/m^2^) until the visualization of at least three follicles ≥17 mm when ovulation was triggered with 250 µg recombinant human chorionic gonadotropin (hCG). In this group 13 women subsequently became pregnant and 16 did not become pregnant (retrospective information). Of those who became pregnant (n = 13), 8 underwent in vitro fertilization (IVF) while 5 underwent intracytoplasmic sperm injection (ICSI). Within this group 2 women had endometriosis, 4 women had polycystic ovary (PCO) and 2 women had polycystic ovarian syndrome (PCOS). Of the women who did not become pregnant (n = 16), 1 underwent IVF and 15 underwent ICSI. Within this group, 3 women had PCO.

In the GnRH antagonist group (GnRH-ant, n = 14) ovarian stimulation was commenced on day 2 of the menstrual cycle with 125–225IU recombinant FSH daily as above until the visualisation of at least one follicle ≥14 mm when administration of GnRH antagonist, cetorelix acetate (0.25 mg), commenced up to and including the day of hCG administration. When at least three follicles ≥17 mm were visualized, ovulation was triggered with 250 µg recombinant hCG. In this group, 6 women underwent IVF while 8 underwent ICSI. Within this group, 2 women had endometriosis. No women in this cohort became pregnant in the cycle of sampling.

In the GnRH agonist oocyte donor group (n = 14), fertile women were stimulated via the GnRH agonist protocol (described above) for oocyte donation. These women followed the same protocol as infertile women until the day of hCG administration and oocyte retrieval. These women did not receive luteal phase progesterone.

Oocyte retrieval was performed two days after hCG administration (hCG+2) by transvaginal ultrasound guided aspiration. Endometrial biopsies were taken by Pipelle on the day of oocyte collection from GnRH agonist donor cycles, GnRH agonist stimulated infertile women and GnRH antagonist stimulated infertile women. All infertile women from whom a biopsy was obtained underwent a fresh embryo transfer with embryos generated from their own oocytes. Fertile oocyte donors did not undergo an embryo transfer, their hormone treatment ceased at ovulation triggering and their study involvement ceased at endometrial biopsy. The luteal phase of all stimulated embryo transfer cycles was supplemented with vaginal administration of 8% progesterone gel (Crinone, Serono) from day 3 after oocyte collection.

Human endometrium was obtained by curettage from normal cycling women 2 days after the LH surge (LH+2: as assessed by urinary assay) following laparoscopic sterilization or assessment of tubal patency (fertile; n = 9). Menstrual cycle stage in normal cycling women was confirmed by histological dating, according to the criteria of Noyes *et al* [30].

All tissue was fixed in formalin and processed to wax under standardized conditions in the same laboratory.

### Histology

Paraffin sections (5 µm) were dewaxed in Histosol (Sigma Chemical Co; St Louis, MO) and rehydrated through descending grades of alcohol (95–70%) to distilled water (dH_2_O). Tissue histology was assessed following hematoxylin and eosin staining.

### Immunostaining

Leukocytes. Leukocyte subpopulations were identified in endometrial tissue using antibodies raised in mouse directed against leukocyte cell markers: total leukocytes (CD45, leukocyte common antigen, LCA, Dako), uterine natural killer cells (uNK) (CD56 Zymed; San Francisco, CA), macrophages (CD68, Dako), and neutrophils (NE, neutrophil elastase), Dako) as described previously [31]. In brief, following deparaffinization and rehydration (as above), sections were subjected prior to staining to: a) antigen microwave retrieval in 0.1 M citrate buffer for CD56 b) trypsin (Sigma) digestion (0.2% in 0.2% calcium chloride/TBS, 10 min at 37°C) for CD68 c) no antigen retrieval for CD45 or NE. Endogenous peroxidase activity was quenched with 3% H_2_O_2_ in dH_2_O for 10 min at room temperature. Non-specific binding was blocked with non-immune blocking solution (10% normal horse serum (H0146, Sigma), 2% normal human serum (‘in-house’) in TBS/0.1% Tween 20 (Bio-Rad, North Ryde, NSW, Australia) in a humidified chamber for 30 min at room temperature. Primary antibodies were subsequently applied overnight (16–18 h) at 4°C, at the following concentrations; CD56 (1.1 µg/ml), CD68 (2.4 µg/ml), CD45 (1 µg/ml) and NE (1.3 µg/ml). Following washing to remove excess antibody (TBS/0.1% Tween 20), biotinylated horse anti-mouse IgG (Vector Laboratories, Burlingame, CA, USA) diluted in non-immune blocking solution (1∶200) was applied for 30 min. Following stringent washing in TBS-Tween (0.6%), antibody localization was detected using an avidin/biotin peroxidase detection system (ABC-HRP, Dako, Botany, NSW, Australia). Positive localization of leukocytes (CD45, CD56, CD68 and NE) was identified by the application of the peroxidase substrate 3, 3′-diaminobenzidine (DAB, Dako), which produces a brown precipitate. Tissue sections were counterstained with hematoxylin, dehydrated through ascending grades of ethanol (70–95%) and histosol and mounted with DPX. Negative controls were included for each tissue section with substitution of the primary antibody with a matching concentration of mouse IgG (mIgG, Dako) as appropriate.

#### Progesterone Receptor

PR protein localization was performed following the immunostaining protocol used for CD56, with substitution of the primary antibody by mouse anti-PR (detecting PR-A plus PR-B) applied at 3.6 µg/ml (Novocastra, North Ryde, Australia). Biotinylated horse anti-mouse IgG was applied at 1∶200 for one hour prior to detection with ABC-HRP and 3, 3′-diaminobenzidine.

#### Vasculature (CD34)

CD34 protein localization was performed following the antigen retrieval protocol used for CD56. Sections were blocked with 10% normal rabbit serum, 2% normal human serum (‘in-house’) in TBS/0.1% Tween 20 for 30 min at room temperature. Goat anti-CD34 was applied at 0.5 µg/ml (Serotec) overnight at 4°C. Biotinylated rabbit anti-goat secondary antibody was applied at 1∶200 for one hour prior to detection with ABC-HRP and 3, 3′-diaminobenzidine.

#### Decidualization marker (prolactin)

No antigen retrieval was performed prior to immunodetection with rabbit anti-prolactin (Dako,N1549) applied at a 1∶1.75 dilution overnight at 4°C in a 10% normal goat serum, 2% normal human serum, TBS/0.1% Tween 20 blocking solution. Biotinylated Goat anti-rabbit (Vector, BA-1000) was applied at 1∶200 for 30 mins prior to detection with ABC-HRP and 3, 3′-diaminobenzidine.

### Analysis of Tissue Histology and Immunostaining

Histological and immunohistochemical examination was performed for tissue structure, blood vessel integrity and localization, PR and changes to immune cell populations using an Olympus CH30 microscope. High-resolution images were captured with a Fujix Hc-2000 digital camera. Tissue histology and positive immunostaining was semi-quantitatively scored by two independent experienced observers blind to the nature of the tissue.

We applied a histological scoring system based on the similarity of the tissues to the appearance of normally fertile tissues at LH+2 (normal according to Noyes criteria [30]). A normal appearance of glandular epithelium (small, minor secretory changes including some vacuoles), stroma (compact) and blood vessels was allocated a score of 2. Tissues which differed from LH+2, with early secretory changes (eg. presence of vacuoles in >50% of endometrial glands, stromal oedema, blood vessel transformation/enlargement) were allocated a score of 1. Tissues which differed more substantially from LH+2 (eg. extensive evidence of glandular secretions, extensive stromal oedema or decidual changes, large expanded blood vessels) were allocated a score of 0.

For assessment of immunohistochemical staining, the amount and intensity of immunostaining within each cellular compartment (glandular and luminal epithelium, stroma) was analyzed and allocated a score from 0–3∶0 (no staining); 1 (minimal staining); 2 (strong staining); 3 (intense staining). Leukocyte numbers within the endometrium were semi-quantitatively scored, using a scoring system based on the proportion of the stroma occupied by leukocytes (0–100%).

### Statistics

All semi-quantitative data is presented as mean ± SEM and was tested for normal distribution. Statistical analysis was performed using ANOVA followed by a Mann Whitney test to determine comparisons between groups. P value <0.05 was taken as significant.

## Results

### Patient Demographics

The data for patient demographics is presented in Table 1. The GnRH antagonist group was significantly older than all other stimulated groups (p<0.05). The BMI for the GnRH antagonist group was significantly lower than the donor agonist and the GnRH agonist not pregnant groups (p<0.05). The peak estrogen (pg/ml) and number of oocytes collected were significantly higher in the GnRH agonist pregnant group compared with the GnRH antagonist and the GnRH agonist not-pregnant groups (p<0.05). The cumulative FSH, parity and previous live births did not significantly differ between the groups examined.

### Endometrial Histology

**Figure 1:**
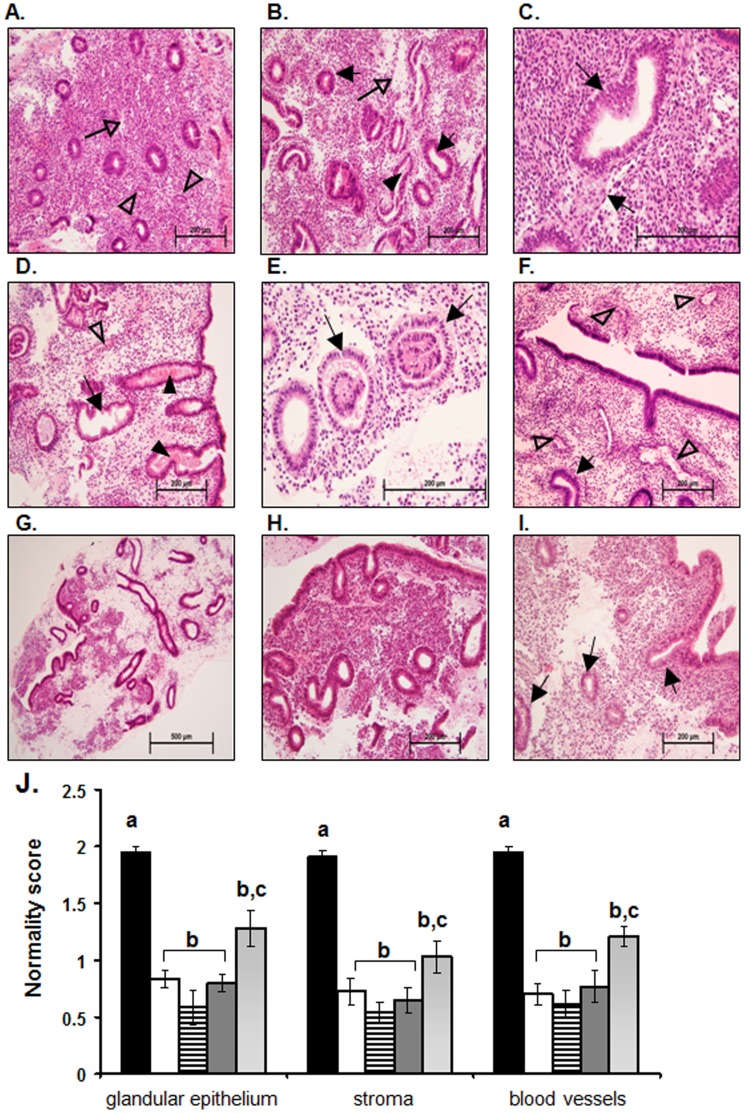
Endometrial histology of IVF stimulated and non-stimulated endometria. In the fertile non-stimulated endometrium at LH+2 (A) the glands are small and straight with only very early signs of secretory changes. The stroma is compact with minor signs of oedema (open arrow), and blood vessels are small (open arrow head). At hCG+2 in the fertile donor endometrium stimulated with GnRH agonist, (B & C) the glandular epithelial cells show secretory changes as evidenced by sub-nuclear vacuoles (C, closed arrows), some evidence of secretory activity can also be observed within the lumen of some endometrial glands (B, closed arrowhead). The stroma is expanded and oedematous (B, open arrow). At hCG+2 in the infertile endometrium stimulated with the GnRH antagonist protocol (D & E) a range of different endometrial cycle stage appearances presented. In some endometria (D) the endometrial glandular epithelial cells have lost their sub-nuclear vacuoles and are highly secretory (D, closed arrowheads) with glands becoming tortuous (D, arrow). The stroma is also highly oedematous and large open blood vessels are present (D, open arrowhead). Other infertile GnRH antagonist stimulated endometria (E) present with sub-nuclear vacuoles evident in the endometrial glands (E, arrows) and expanded stroma. In the endometria at hCG+2 of infertile women stimulated with the GnRH agonist protocol who did not subsequently become pregnant (F & G) a mixed picture of endometrial histology is observed. Sub-nuclear vacuoles are present in the endometrial glandular epithelial cells (F, closed arrow) with the presence of expanded oedematous stroma and large expanded blood vessels close to the luminal epithelium (F, open arrowheads). In other endometria from this group (G) the tissue histology is disturbed with highly developed, tortuous, secretory endometrial glands and highly oedematous stroma, with the tissue as a whole presenting a fragile appearance. In infertile women stimulated with the GnRH agonist protocol who subsequently became pregnant (H & I) fairly small compact endometrial glands with only early signs of secretory transformation (I, arrows) were observed at hCG+2. The stroma, while expanded in some areas is generally compact and the blood vessels are not highly developed. Endometrial histology was scored by means of a ‘normality score’ (J) with tissues of normal histology for LH+2 allocated a score of 2, tissues with somewhat changed histology allocated a score of 1 and tissues with highly altered histology allocated a score of 0. Data for fertile women are presented in black (?), data for fertile donors are presented in white (□), data for infertile GnRH antagonist are presented in horizontal lines (≡), data for infertile GnRH agonist not pregnant are presented in dark grey (?), data for infertile GnRH agonist pregnant are presented in light grey (?). Data are presented as mean ± SEM. a is significantly different from b, p<0.01; b is significantly different from c, p<0.05.

Histologically, the non-stimulated fertile endometrium at LH+2 (n = 9) presented with normally organized narrow, straight glands containing no or very few vacuoles (Figure 1A). The glandular structures were not significantly altered in appearance from those typically observed in the late-proliferative/early-secretory phase of the menstrual cycle [30], [32]; however some did show minor early secretory changes, such as presence of vacuoles, as expected. The stroma was undifferentiated and compact with minimal evidence of edema (Figure 1A, open arrow) as expected for this phase of the cycle.

Examination of GnRH agonist-stimulated endometria from fertile oocyte donor cycles revealed extensive sub-nuclear vacuoles within the glandular epithelial cells (Figure 1B & 1C, arrows). The glands were highly developed and tortuous with increased diameter and prominent signs of secretory activity within the gland lumen (Figure 1B, arrowhead). Stromal edema was also evident (Figure 1B, open arrow). Semi-quantitative analysis showed that development of the glandular epithelium, the stroma and the blood vessels within endometria from donor cycles were significantly different compared with normal fertile endometria at LH+2 (Figure 1J, P<0.001).

Endometrial tissues from infertile women stimulated with the GnRH antagonist protocol generally presented with highly tortuous, secretory glands (Figure 1D, arrows and arrow heads respectively) containing extensive sub-nuclear vacuoles (Figure 1E, arrows). The stromal compartment was expanded and edematous (Figure 1D). Enlarged blood vessels (Figure 1D, open arrow head) were also noted. Semi quantitative analysis demonstrated significantly different endometrial histology of infertile GnRH antagonist subjects compared with normal fertile endometria at LH+2 (Figure 1J, P<0.001).

Infertile women stimulated with the GnRH agonist protocol were subdivided into two groups; those who did or did not become pregnant following the biopsy, allowing correlation of histological changes with pregnancy outcome.

Endometrial samples from women who did not become pregnant were heterogeneous in appearance. Some had a secretory phenotype, with sub-nuclear vacuoles present in the glandular epithelium (Figure 1F, arrows), oedematous stroma and enlarged blood vessels close to the luminal epithelial surface (Figure 1F, open arrow heads). Other tissues had a mixed appearance with highly developed tortuous, secretory glands adjacent to small compact glands (Figure 1G) and with strongly edematous stroma (Figure 1G). These ‘mixed-appearance’ tissues appeared to be highly fragile, likely due to the degree of edema. Semi quantitative analysis demonstrated the histological appearance of these endometria was significantly different compared with normal fertile endometria at LH+2 (Figure 1J, P<0.001).

In women stimulated with GnRH agonist who subsequently became pregnant, the endometrial histology was less disturbed than in those who did not become pregnant. The glands were generally smaller and more compact (Figure 1H and 1I). However, some glands did present signs of secretory advancement with sub-nuclear vacuoles present (Figure 1I, arrows), but no evidence of secretions within the lumen. Some stromal oedema was observed (Figure 1I), but this was less than in women who did not become pregnant (Figure 1G). Semi quantitative analysis demonstrated these endometria were significantly different compared both with normal fertile endometria at LH+2 (Figure 1J, P<0.001) and with other stimulated groups (Figure IJ, P<0.05).

Thus, the histological appearance of the endometrium in the ‘pregnant’ group represents an ‘intermediate’ between the substantially altered histological appearance of the other stimulated groups (fertile donor, infertile antagonist, and infertile agonist not pregnant) and the normal fertile endometrium at LH+2.

### Progesterone Receptor

**Figure 2:**
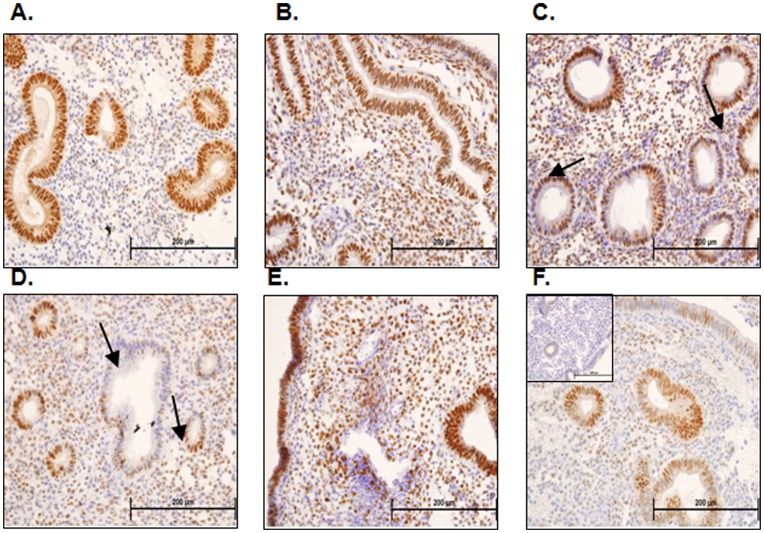
Progesterone receptor immunohistochemistry and semi-quantitative analysis. Progesterone receptor (PR) consistently immunolocalizes to the glandular epithelial cells of endometria taken from fertile women at LH+2 (A). In fertile ovum donors stimulated with the GnRH protocol PR immunostaining is localized to glandular epithelial cells, luminal epithelial cells and stromal cells on hCG+2 (B). PR staining is similarly patchy in the glandular epithelial cells of endometria taken from infertile women stimulated with GnRH antagonist at hCG+2 (C & D, arrows) and in the stroma (C & D). PR immunostaining localizes to the glandular epithelial cells, luminal epithelial cells and stromal cells of endometria of infertile women undergoing the GnRH agonist protocol who did not subsequently become pregnant at hCG+2 (E). PR immunolocalized mainly to the glandular epithelium with patchy luminal epithelial and stromal staining in endometria of infertile women undergoing the GnRH agonist protocol who subsequently become pregnant at hCG+2 (F).

Immunoreactive PR showed no significant changes upon semi-quantitative analysis, although trends were apparent in staining of luminal epithelium, glands and stroma of stimulated (Figure 2B–F) versus normal fertile tissues at LH+2 (Figure 2A).

### Leukocytes

**Figure 3:**
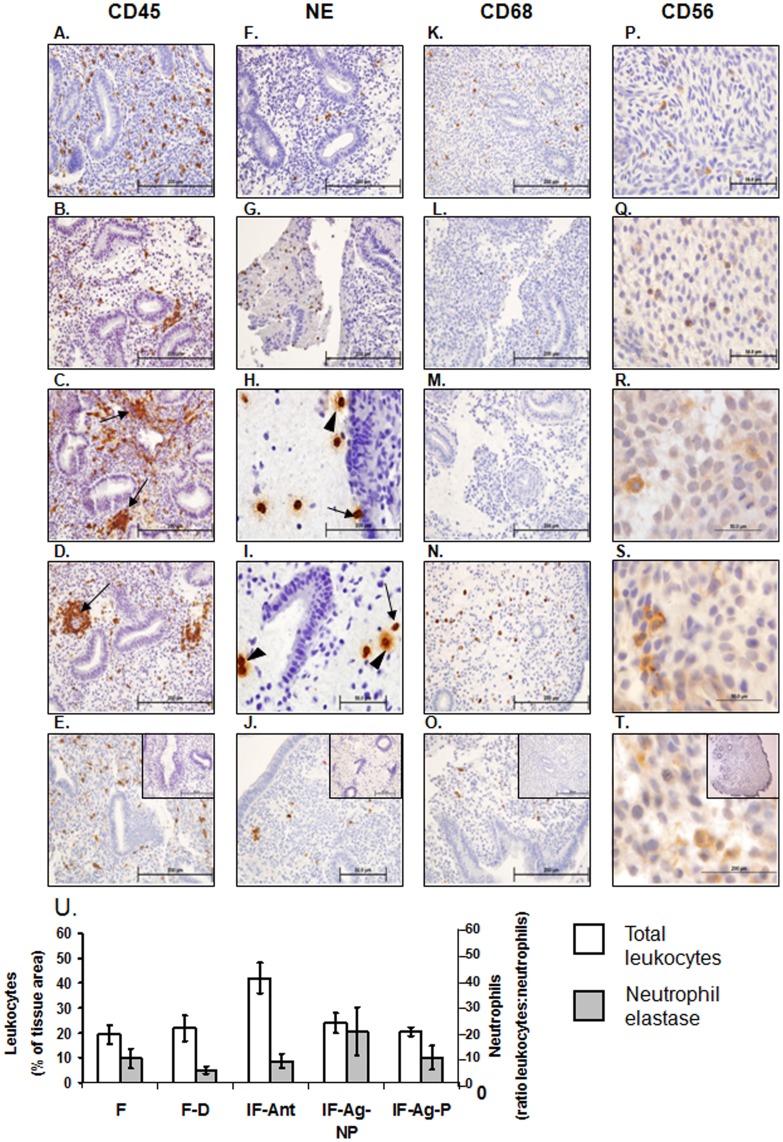
Leukocyte immunohistochemistry and semi quantitative analysis. Immunohistochemical staining was performed to examine the localization leukocyte subtypes (CD45/leukocyte common antigen, A-E), neutrophils (neutrophil elastase, F-J), macrophages (CD68, K-O) and uterine natural killer cells (CD56, P-R). In fertile endometria at LH+2 leukocytes are scattered throughout the tissue (A). At hCG+2 in stimulated endometria from fertile donor women undergoing GnRH agonist stimulation (B), infertile women undergoing GnRH antagonist stimulation (C) and infertile women undergoing GnRH agonist stimulation who did not become pregnant (D) clusters of leukocytes can be observed particularly adjacent to endometrial glands and surrounding blood vessels (B, C, and D, arrows). In endometria of women stimulated with the GnRH agonist protocol who subsequently became pregnant at hCG+2 (E) leukocytes are found scattered throughout the tissue, similar to that observed in fertile women at LH+2 (A). Very few neutrophils can be observed in the endometria of fertile women at LH+2 (F) or fertile women undergoing GnRH agonist stimulation for ovum donation at hCG+2 (G). In stimulated endometria from GnRH antagonist stimulated infertile women (H) or GnRH agonist protocol who did not become pregnant (I) numerous neutrophils can be observed particularly within mucous like areas and to a lesser degree within the tissue. Many of these are degranulating (H & I, closed arrowhead). Very few neutrophils were evident in the endometria of GnRH agonist stimulated infertile women who became pregnant (J). Scattered macrophages could be observed in endometria of fertile women at LH+2 (K). Very few macrophages were present in fertile women undergoing GnRH agonist stimulation for ovum donation (L) or infertile women undergoing GnRH antagonist stimulation (M). Scattered macrophages were observed in the endometria of infertile women stimulated with the GnRH agonist protocol whether they did not (N) or subsequently achieved (O) pregnancy. Few uterine natural killer cells were detected at LH+2 (P) or hCG+2 in endometria of fertile (Q) infertile women stimulated with the GnRH antagonist protocol (R), or the GnRH agonist protocol who did not (S) or did (T) become pregnant at hCG+2. IgG controls are shown inset in panels E, J, O and T. Scale bars are shown in each image, all 200 µm except P - T at 50 µm. Semi quantitative scoring of total leukocyte numbers (U) revealed increased leukocyte numbers in endometria of infertile women stimulated with the GnRH antagonist protocol at hCG+2 compared with fertile women at LH+2 (U, white bars, P<0.05). No significant differences in neutrophil numbers were observed (U, grey bars). F = fertile, IF = infertile, F–D = fertile donor, IF-Ant = infertile antagonist, IF-Ag-NP = infertile agonist non-pregnant, IF-Ag-P = infertile agonist pregnant. Data are expressed as mean ± SEM, *P<0.05.

Total leukocyte numbers (CD45+) were significantly elevated (Figure 3U, P<0.05) in endometria of infertile women stimulated with antagonist (Figure 3C) compared with fertile women at LH+2 (Figure 3A, 21.0±4.4% (fertile) vs 39.1±6.6% (antagonist), Figure 3U), but did not change significantly in other groups. However, altered leukocyte localization was observed, with CD45+ cells clustered around endometrial glands and blood vessels in the stimulated endometria (Figure 3C & 3D) rather than scattered throughout the tissue as observed at LH+2 (Figure 3A).

Total neutrophil number (detected by staining for neutrophil elastase) did not alter significantly with treatment (Figure 3U) but both their localization and activation state were changed in stimulated endometria. Neutrophils congregated in the edematous areas of stimulated endometria (Figure 3H & 3I) with many clearly undergoing degranulation, (release of their cytotoxic intracellular contents, Figures 3H & 3I, arrows). Localization and numbers of CD68+ macrophages (Figure 3K–O) and CD56+ uNK cells (Figure 3P–T) did not differ between groups.

### Vasculature

**Figure 4:**
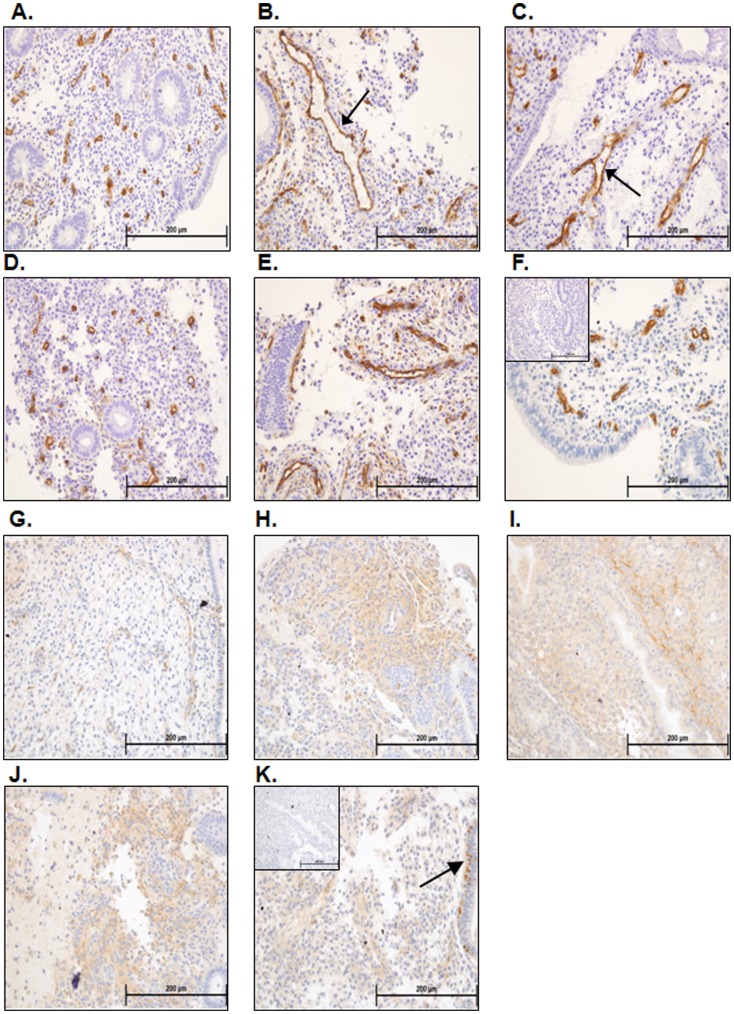
Vasculature/CD34 and decidualization/prolactin immunostaining. Blood vessels within the endometria of fertile women at LH+2 were generally small and compact (A). Examination of endometria from fertile women stimulated with the GnRH agonist protocol for ovum donation at hCG+2 revealed a mixture of small blood vessels and grossly enlarged blood vessels (B, arrow). Endometria from infertile women stimulated with the GnRH antagonist protocol at hCG+2 revealed a mixed picture of vascular development, with some endometria presenting grossly enlarged blood vessels (C, arrow) whereas some presented with small compact blood vessels (D). Women stimulated with the GnRH agonist protocol at hCG+2 who did not subsequently become pregnant presented with large endometrial blood vessels (E), whereas women stimulated with this protocol who did become pregnant generally presented with smaller more compact blood vessels (F). Fertile women at LH+2 had little to no immunostaining for prolactin within the endometrial stromal compartment (G). Some areas within the endometria of fertile women stimulated with the GnRH protocol for ovum donation (H), infertile women stimulated with the GnRH antagonist protocol (I) and infertile women stimulated with the GnRH agonist protocol who did not become pregnant (J) at hCG+2 demonstrated intense stromal prolactin immunostaining indicative of decidual changes. Endometria from infertile women stimulated with the GnRH agonist protocol at hCG+2 who subsequently became pregnant (K) demonstrated little stromal prolactin immunostaining, but did demonstrate glandular prolactin immunostaining indicative of secretory changes (K, arrows).

Endometrial blood vessels were assessed using CD34 staining for endothelial cells (Figure 4A–F). Fertile women at LH+2 presented with mainly small vessels, as expected at this stage of the menstrual cycle (Figure 4A). Stimulated endometria generally presented with blood vessels which appeared enlarged regardless of fertility status or stimulation protocol. In GnRH- antagonist stimulated infertile women, blood vessels were heterogeneous, being either enlarged (Figure 4C, arrows), or small as in fertile women at LH+2 (Figure 4A vs 4D). In agonist-stimulated women who did not become pregnant, enlarged blood vessels were observed immediately below the endometrial surface (Figure 4E). In agonist-treated women who did become pregnant, the blood vessels were mainly similar in appearance to those in fertile women at LH+2 (Figure 4F vs 4A).

### Decidualized Stromal Cells

Decidualization of the stroma, demonstrated by prolactin immunostaining, is initiated during the mid-late secretory phase in natural menstrual cycles, specifically close to spiral arterioles. In non-stimulated tissues (LH+2) of fertile women, the stroma was compact with little evidence of edema or prolactin staining (Figure 4G). Stimulated tissues at hCG+2 were edematous (Figure 4H –4K) with apparent pre-decidualized stromal cells around the spiral arterioles and in the sub-epithelial region: these were immunopositive for prolactin. However, the extent of stromal decidualisation was variable (Figure 4H –4K).

## Discussion

In assisted reproduction an embryo that has been fertilized in vitro is replaced into the uterine cavity of the women where it can implant and establish a pregnancy. However, the hormonal regimen used to stimulate ovum development earlier in the menstrual cycle, may disturb endometrial development such that it is not fully receptive for implantation. This paper defines substantial changes in endometrial histoarchitecture and immunocytochemical markers of endometrial differentiation induced by ovarian stimulation protocols in IVF treatment cycles and, importantly, has shown that these disturbances were less severe in women who subsequently become pregnant than in those women who did not. It also demonstrates significant differences in the localization, activation status and number of leukocytes in the endometria of infertile women stimulated for ART, suggesting an enhanced inflammatory environment. This supports and extends previous evidence that the ART endometrium represents an inherently altered tissue when compared with endometria from the natural cycle at an equivalent cycle stage and that this is not conducive to the establishment of pregnancy. While the accuracy and reliability of standard methods for assessing and dating the endometrium based on subtle changes in endometrial histology, has been seriously challenged in a number of studies [33], [34] the histological changes observed here between fertile non-stimulated versus stimulated endometria were obvious. Further, this study applied not only histological examination but also immunohistochemistry, examining markers of cellular differentiation.

Previous studies have focused mainly on only one IVF protocol making it difficult to interpret the overall ART protocol effects on endometrial histology. Disparities can arise from the stimulation protocol, the patient selection criteria and the endocrinological parameters [20]. In this study, inclusion of endometria from women undergoing stimulation via GnRH agonist and antagonist protocols allowed us to examine differences likely caused by protocol effects. Comparison of fertile non-stimulated subjects with previously fertile oocyte donors stimulated via the GnRH agonist protocol, enabled assessment of the effect of this protocol on endometrial histology without the confounding influence of infertility. We did not apply stringent selection criteria with respect to age or BMI, which may present confounding influences in the overall interpretation of this study. However, while a detrimental effect of obesity has been demonstrated on oocyte quality [35], no such effect has been demonstrated thus far for the endometrium. It may also be questioned whether an endometrial biopsy may represent a confounding influence on pregnancy outcome. However, all infertile women who underwent stimulation for IVF with a biopsy taken on the day of oocyte retrieval subsequently underwent a fresh embryo transfer. While we cannot anticipate the local inflammatory response subsequent to biopsy for each individual woman, sampling of every participant controlled for any effect of biopsy.

Initial comparisons between normally fertile women at LH+2 and fertile women undergoing agonist stimulation for ovum donation demonstrated significant endometrial histological changes. We propose these changes are mediated by the GnRH agonist protocol, which by facilitating multi-follicular development, exposes women to supra-physiological levels of FSH and a higher level of estrogen, in addition to precocious rises in progesterone if ovulation is not appropriately suppressed [18], [24], [36], [37], [38]. The observed alterations in histology are therefore likely to result from the rapid rise and higher levels of these hormones or possibly more directly by the GnRH agonist (Synarel) itself [39].

The infertile GnRH antagonist group at hCG+2 presented a highly heterogeneous collection of samples. While some tissues showed extremely advanced endometria (Figure 1D), others had more modest secretory changes (Figure 1E). The GnRH antagonist protocol, combined with a GnRH agonist for ovulation induction, is now considered by some clinics as a better option for ART due to the reduced risk of ovarian hyperstimulation syndrome (OHSS). However, these protocols have a detrimental effect on endometrial quality [40]. The significant advancement of the endometria observed in this study reinforces the issue of compromised endometrial quality.

The infertile GnRH agonist group at hCG+2 was divided depending on pregnancy outcome. Importantly, while still altered compared with normal fertile women at LH+2, the group who became pregnant had significantly less disturbed endometrial histology than those who did not become pregnant: the latter showed features of secretory changes and evidence of a ‘fragile’ edematous endometrium, such as that often observed in women using hormonal contraceptives [41], [42]. The non-pregnant group also had large open blood vessels close to the endometrial surface (Figure 1F) and highly edematous endometrial stroma with secretory,tortuous endometrial glands (Figure 1G). In contrast, the endometria of the pregnant group appeared more intact, with smaller tighter glands, similar to those observed at LH+2 in the natural cycle, and quantitatively fewer histological changes than in the non-pregnant group. Since late implantation into a late secretory endometrium (>10 days after ovulation) correlates with an increased risk of early miscarriage [43] it is likely that implantation into the advanced and out-of-phase endometrium also results in very early miscarriage, prior to pregnancy testing. In support of this, we demonstrate that pregnancy is only achieved in the presence of a ‘significantly less disturbed’ endometrium.

In this study we examined progesterone receptors as previous studies have demonstrated significant differences in progesterone receptors between fertile women and those stimulated for IVF via the GnRH antagonist protocol [27], [44] in the absence of histological changes. Interestingly in this study we observed the opposite pattern, no change in progesterone receptors but a significant change in endometrial histology. The major differences between the two studies are 1) the day of sampling, with endometrial biopsies taken on the day of ovulation trigger in the Papanikolaou study (hCG+0) [44] and biopsies taken on the day of oocyte retrieval (hCG+2) in this study, and 2) the study population, with a heterogeneous population examined in this study and a matched sample taken from the same women in a natural cycle and a subsequent stimulated cycle in the Papanikolaou study. In the present study we are likely to be observing an effect of both hCG on endometrial histology, and of precocious progesterone rises resulting in secretory transformation of the endometrium on this later cycle day (hCG+0 vs hCG+2). The lack of quantifiable difference in progesterone receptor may be due to the heterogeneity of the sample population.

Endometrial leukocytes undergo dramatic changes in both number and relative composition throughout the normal menstrual cycle. Whereas leukocytes are present in relatively low numbers during the proliferative phase of the cycle, these increase through the secretory phase until peri-menstrually they account for around 40% of the cell content of the stromal compartment [45].Uterine NK cells and macrophage numbers increase in association with receptivity, whereas both numbers and activation of neutrophils increase significantly only prior to menstruation [45]. In agreement with published data, we show that stimulation for IVF increases total endometrial leukocyte numbers [46]. Importantly, we present the first evidence for an increase in endometrial neutrophil numbers and activation status, as demonstrated by degranulation, in women stimulated with either antagonist or agonist protocols, who did not become pregnant. Neutrophil activation (normally seen at menstruation) releases inflammatory mediators including protease enzymes and chemokines that mediate leukocyte recruitment and blood vessel leakiness, likely contributing to the stromal oedema noted within these tissues. In contrast, the immune cell populations in endometria of the women who became pregnant were similar to those in fertile women at LH+2. Thus, the endometrial fragility induced by neutrophil activation may well contribute to the failure of some women to establish pregnancy. Our observed lack of changes in endometrial uNK cell numbers in the stimulated endometrium is in agreement with the data of Lukassen et al [47] who demonstrate that IVF does not influence uNK cell numbers relative to total CD45 positive cells. While uNK cells are proposed to facilitate decidualization and implantation [48] no differences in their numbers at hCG+2 were observed in the present study between women who did or did not become pregnant. Although dendritic cells and T-regulatory cells play a major role in the implantation process [49], [50], [51] since their numbers do not change significantly in the functionalis across the normal menstrual cycle [52], [53] we did not examine their numbers in this study. It should be appreciated, however, that the activation status of these cells can dramatically influence other endometrial cells including other leukocytes within the microenvironment. As this was a retrospective study we did not have access to fresh tissue from which these cells could be isolated and examined further.

Pre-decidual cells are normally present in cycling endometrium, only in the late secretory phase [54]; these are positively identified here by prolactin staining in all stimulated tissues at hCG+2, indicating that the endometrial stromal compartment is advanced to an equivalent of LH+9/10. This is consistent with the high degree of glandular-stromal dys-synchrony previously reported in ART endometrium [55]. Importantly, few decidual changes were observed in the stromal compartment of the subjects stimulated with the GnRH agonist protocol who subsequently became pregnant, indicating that in-phase glandular-stromal development is important for a positive pregnancy outcome.

It is thus clear that ART endometria at hCG+2, regardless of stimulation protocol or previous fertility status, demonstrate altered endometrial development and that the extent of this disturbance can determine whether or not pregnancy can be established in that cycle. This study does not aim to provide a predictive histological test for endometrial receptivity as the biopsy, tissue processing and immunohistology required are burdensome and unlikely to provide an indication of whether an embryo should be placed into the uterine cavity within the same cycle. Quick assessments of uterine receptivity markers present in a minimally invasive uterine lavage sample [3], [4], [5], [6], [11] are more likely to provide timely indications of receptivity.

It is possible that modifications to current clinical ART stimulation protocols may provide more normal synchronous endometrial development. Recently developed ‘low intensity’-IVF stimulation protocols make controlled ovarian hyperstimulation less complex, cheaper and with reduced chances for adverse complications [56]. These involve the use of clomiphene citrate from day 3 of the cycle and a low level of human menopausal gonadotropin (150IU) for ovarian development with a GnRH agonist used for triggering ovulation [57] with the aim of developing a maximum of 8 follicles. This contrasts with the extended stimulation protocol described herein (14 days with GnRH agonist followed by FSH and ovulation trigger with hCG). However, the efficacy of low intensity IVF procedures has been called into question [58], [59] and their effect on endometrial development has not yet been investigated. A more appropriate change, from the findings of this study, would be cryopreservation followed by replacement into a natural menstrual cycle. Recent developments and optimization of vitrification and thawing techniques have demonstrated that improved pregnancy rates are possible with transfer of cryo-preserved thawed embryos into a natural cycle [9], [10] dated by the natural LH surge (compared with cycles where ovulation was triggered by hCG (31.1% vs 14.3% pregnancy respectively, [10]). From the combined evidence available, cryopreservation of embryos and transfer into a natural cycle may assist in improving IVF outcomes.

In conclusion, this paper demonstrates considerable disturbance of endometrial histology in women stimulated for ART, irrespective of fertility status. Importantly, in those infertile women who did become pregnant following stimulation, the endometrium more closely resembled the fertile endometrium at LH+2. We propose that the disturbed endometrial transformation and leukocyte activation status defined here, render the endometrium less or non receptive for embryo implantation, indicating that either modification of protocols or transfer of frozen embryos into natural cycles, may help optimise outcomes for infertile couples.
